# Agronomic advantage of bacterial biological nitrogen fixation on wheat plant growth under contrasting nitrogen and phosphorus regimes

**DOI:** 10.3389/fpls.2024.1388775

**Published:** 2024-05-08

**Authors:** Abderrahim Aasfar, Issam Meftah Kadmiri, Salah Eddine Azaroual, Sanaâ Lemriss, Najib El Mernissi, Adnane Bargaz, Youssef Zeroual, Abderraouf Hilali

**Affiliations:** ^1^ Plant and Microbial Biotechnology Center, Moroccan Foundation for Advanced Science, Innovation and Research (MAScIR), Mohammed VI Polytechnic University, Ben Guerir, Morocco; ^2^ Laboratory of Health Sciences and Technologies, High Institute of Health Sciences, Hassan 1st University, Settat, Morocco; ^3^ Department of Biosecurity PCL3, Laboratory of Research and Medical Analysis of Gendarmerie Royale, Rabat, Morocco; ^4^ AgroBioSciences, College of Agriculture and Environmental Sciences, Mohammed VI Polytechnic University, Ben Guerir, Morocco; ^5^ Situation Innovation Group–Office Chérifien des Phosphates (OCP Group), Jorf Lasfar, Morocco

**Keywords:** nitrogen fixation, phosphorus, wheat, *Rhodotorula mucilaginosa*, *Arthrobacter* sp

## Abstract

**Introduction:**

Given their remarkable capacity to convert atmospheric nitrogen into plant-accessible ammonia, nitrogen-fixing microbial species hold promise as a sustainable alternative to chemical nitrogen fertilizers, particularly in economically significant crops like wheat. This study aimed to identify strains with optimal attributes for promoting wheat growth sustainably, with a primary emphasis on reducing reliance on chemical nitrogen fertilizers.

**Methods:**

We isolated free nitrogen-fixing strains from diverse rhizospheric soils across Morocco. Subsequently, we conducted a rigorous screening process to evaluate their plant growth-promoting traits, including nitrogen fixation, phosphate solubilization, phytohormone production and their ability to enhance wheat plant growth under controlled conditions. Two specific strains, *Rhodotorula mucilaginosa* NF 516 and *Arthrobacter* sp. NF 528, were selected for in-depth evaluation, with the focus on their ability to reduce the need for chemical nitrogen supply, particularly when used in conjunction with TSP fertilizer and natural rock phosphate. These two sources of phosphate were chosen to assess their agricultural effectiveness on wheat plants.

**Results and discussion:**

Twenty-two nitrogen-fixing strains (*nif-H*+) were isolated from various Moroccan rhizospheric soils, representing *Bacillus* sp., *Pseudomonas* sp., *Arthrobacter* sp., *Burkholderia* sp. and a yeast-like microorganism. These strains were carefully selected based on their potential to promote plant growth. The findings revealed that the application of *Rhodotorula mucilaginosa* NF 516 and *Arthrobacter* sp. NF 528 individually or in combination, significantly improved wheat plant growth and enhanced nutrients (N and P) uptake under reduced nitrogen regimes. Notably, their effectiveness was evident in response to both natural rock phosphate and TSP, demonstrating their important role in wheat production under conditions of low nitrogen and complex phosphorus inputs. This research underscores the significant role of nitrogen-fixing microorganisms, particularly *Rhodotorula mucilaginosa* NF 516 and *Arthrobacter* sp. NF 528, in wheat production under conditions of low nitrogen and complex phosphorus inputs. It showcases their potential to reduce chemical nitrogen fertilization requirements by up to 50% without compromising wheat plant yields. Our study emphasizes the importance of bacterial biological nitrogen fixation in meeting the remaining nitrogen requirements beyond this reduction. This underscores the vital role of microbial contributions in providing essential nitrogen for optimal plant growth and highlights the significance of biological nitrogen fixation in sustainable agriculture practices.

## Introduction

Agricultural productivity is often limited by the availability of soil nutrients, with nitrogen (N) being a key limiting factor. Although N is among the most abundant elements on Earth (making up about 80% of the atmosphere) (Cabello et al., 2009), most organisms cannot use it in its molecular form. Specifically, plants cannot directly assimilate atmospheric nitrogen (N_2_), they absorb the available N in the soil through their roots in the form of ammonium and nitrates (Santi et al., 2013). Therefore, the input of N into the soil for plant nutrition and crop productivity mainly relies on the decomposition of organic matter, the application of synthetic fertilizer and the activity of microbial nitrogenase enzymes through Biological Nitrogen Fixation (BNF) (Vitousek et al., 2013; Aasfar et al., 2021).

The BNF process, which is primarily associated with prokaryotic cells, converts N_2_ into a simple, soluble form that plant cells can use to synthesize various biomolecules. This process is mediated by the nitrogenase enzyme, which is quite similar in most of the N_2_-fixing bacteria (Soumare et al., 2020). Published estimates of the amount of N fixed annually through the BNF process have shown variations, with figures ranging from approximately 1.95 × 10^11^ kg (Galloway et al., 2004), 2.5 × 10^11^ kg (Cheng, 2008).

Bacteria that fix N_2_ into biologically usable ammonium are referred to as diazotrophs. Diazotrophs are found in various ecosystems among archaea and bacteria (Zehr et al., 2003). These bacteria may possess additional beneficial traits for plant growth alongside their primary function of nitrogen fixation. These traits include the production of plant growth-promoting phytohormones (e.g., indole-3-acetic acid, gibberellins), the production of polysaccharides and siderophores, biological control of soil-borne pathogens (Andersen and Winding, 2004), and the production of organic acids that enhance plant growth, fostering a mutually beneficial plant-microbe interaction (Garvin and Lindemann, 1986; Dobbelaere et al., 2003). A diverse array of bacteria, including genera like *Azoarcus*, *Azospirillum*, *Arthrobacter*, *Azotobacter*, *Bacillus*, *Burkholderia*, *Erwinia*, *Enterobacter*, *Gluconacetobacter*, *Herbaspirillum seropedicae*, *Klebsiella*, *Kosakonia*, *Paenibacillus*, *Pantoea*, *Pseudomonas*, *Stenotrophomonas*, *Serratia*, and *Xanthomonas*, are among the main plant growth promoting rhizobacteria used to enhance the growth of several crops (Somers et al., 2004; Bhattacharyya and Jha, 2012; Carvalho et al., 2014; Rafikova et al., 2016; Xing et al., 2016; Solanki et al., 2017).

Plant growth-promoting N_2_-fixing strains have proven to be valuable in agriculture, enhancing the growth and yield of various crops. Extensive studies have illustrated their potential to fix N_2_ through molecular, biochemical methods and by direct plant inoculation under greenhouse and field conditions. Ding et al. (2005) found the *nif-H* gene in both *Bacillus* and *Paenibacillus* genera. Similarly, the genera *Pseudomonas*, *Azospirillum* and *Pantoea* showed the presence of the *nif-H* gene in their genome (Venierak et al., 2011; Chauhan et al., 2015; Li et al., 2017). Szilagyi-Zecchin et al. (2014) reported that endophytic *Bacillus* sp. exhibited N_2_ fixation ability, which was assessed through the acetylene reduction assay. Several bacteria such as *Azospirillum*, *Klebsiella*, *Burkholderia*, *Bacillus* and *Pseudomonas* have been identified as Plant Growth-Promoting Rhizobacteria (PGPR). They benefit plants not only through BNF but also by other means, including phosphate solubilization, phytohormone production and controlling soil pathogens (Chelius and Triplett, 2001; Montañez et al., 2009; Piromyou et al., 2011; Zakry et al., 2012; Arruda et al., 2013). BNF by PGPR has been reported to contribute significantly to the total nitrogen uptake in field crops, with contributions ranging from 12–70% or up to 26.7 kg N ha^−1^ (70% of total N uptake) in crops like maize (Montañez et al., 2009), sugarcane (Boddey et al., 1995) and oil palm (Zakry et al., 2012).

The large-scale application of N_2_-fixing bacteria as biofertilizers has been widely studied, particularly as inoculants for non-legume crops, In Egypt, El-Sayed et al (2014) showed significant increases in barley grain yield after inoculation with biofertilizers, which consisted of a mixture of phosphorus-solubilizing and N_2_-fixing bacteria. In the first season, there was a 24.8% increase, and a 27.2% increase in the second season, compared to non-inoculated plants. These results were comparable to the yield obtained with chemical fertilizers. Rose et al. (2014) demonstrated that plant growth-promoting microorganisms, when formulated as inoculant biofertilizers, could replace 23–52% of chemical nitrogen fertilizers in rice-growing systems in Southeast Asia without any loss in yield. Antunes et al. (2019) showed that inoculation with *Herbaspirillum seropedicae*, *Pseudomonas* sp. and *Bacillus megaterium* increased sugar cane yield by 18% to 57.31%.

Nonetheless, there is currently limited information available on the N_2_-fixing abilities of diazotrophs when used in combination with chemical fertilizers. Therefore, the aim of this study was to isolate, identify, and characterize N_2_-fixing strains native to rhizospheric soils of Morocco. Our focus was on investigating their biological nitrogen fixation and plant growth promoting traits, with the objective of contributing to the reduction of chemical nitrogen fertilizer inputs. The strains were evaluated for their plant growth promotion traits, including N_2_ fixation, P-solubilization, AIA production, and *in vitro* inoculation of wheat plants. Subsequently, most performing strains were retained to assess their potential to reduce the reliance on nitrogen-based chemical fertilizers when applied to wheat plants at various levels of chemical nitrogen input. These evaluations were conducted in combination with a commercial phosphate fertilizer (TSP) and natural phosphate (NP) as a source of phosphorus (P) fertilization. This dual-phosphorus methodology is designed to systematically study the complex relationships between these selected strains and various phosphorus inputs. The deliberate incorporation of rock phosphate is of particular importance due to its characterization as a minimally processed and ecologically sustainable phosphorus reserve.

## Materials and methods

### Collection of rhizospheric soils

A total of 82 rhizosphere and non-rhizospheric soil samples were collected from different agricultural regions in Morocco during the years 2017 and 2018. For rhizospheric soils, the samples were taken from the area within a 10-centimeter radius from the surface of the plant roots. Soil samples were collected from depths ranging between 2 and 20 cm. These samples were placed in sterile containers and immediately transported to the laboratory. To maintain their integrity, the samples were stored at 4°C and processed within 48 hours of collection.

### Isolation of free nitrogen fixing bacteria

From each soil sample, 0.1 g of soil was taken and homogenized in 1 mL of sterile physiological water to create a soil suspension. These soil suspensions were then serially diluted (10^-2^–10^-9^), and aliquots (100µL) were spread evenly on Burk’s agar plates (Reis et al, 1994). The plates were incubated at 30°C for a period of 4-5 days. After incubation, individual colonies selected and transferred onto Burk’s agar plates to ensure their purity. For long-term preservation, each isolate was stored at -80° in Burk’s medium with the addition of 30% (v/v) glycerol. In the screening and characterization experiments of the isolated nitrogen fixing strains, a reference strain, *Azotobacter chroorcoccum* DSM2286, obtained from DSMZ, was used as internal positive control.

### Molecular screening and identification of nitrogen fixing strains

The objective of this molecular screening was to identify the presence of the *nif-H* gene in the DNA of the isolated strains using two pairs of primers specific to the nif gene. The primers used were nif H-g1 as described in Reiter et al. (2003): 5’-GGTTGTGACCCGAAAGCTGA-3’/5’-GCGTACATGGCCATCATCTC-3’. Additionally, the primers Pol-F/Pol-R were used (Gaby and Buckley, 2012): 5’-TGC-GAY-CCS-AAR-GCB-GAC-TC-3’ and 5’-ATS-GCC-ATC-ATY-TCR-CCG-GA-3’.

Bacterial strains that were found to have the *nif-H* gene in their genome were identified based on their partial 16S ribosomal DNA (16S rDNA) sequences. The genomic DNA from the bacterial strains was extracted using the PureLink™ Genomic DNA Mini Kit (Invirogen, K182001). PCR reactions were performed using DreamTaq DNA Polymerase PCR Master Mix with the following components: 1 μg of extracted DNA, 0.4 mM dNTPs, 1 μM of each primer, 4 mM MgCl_2_ (Invirogen, K1071), in a final reaction volume of 25 μL. The primers 27F (5′-AGA GTT TGA TCC TGG CTC AG-3′) and 1492R (5′- ACG GTT ACC TTG TTA CGA CTT-3′) (Gauri et al., 2009), were used to amplify the 16S rDNA. The thermocycling conditions involved an initial denaturation at 95°C (1–3 min), followed by 35 cycles of 95°C for 30 s, 53°C for 30 s and 72°C for 1 min, with a final extension at 72°C for 15 min. The PCR products were confirmed on a 1% agarose gel and purified using PureLink Quick Gel Extraction Kit (Invitrogen, K220001). Subsequently, the PCR products were sequenced at Secugen S.L. (https://www.secugen.es ) and the obtained sequences were compared with those available in the NCBI server (https://blast.ncbi.nlm.nih.gov/Blast.cgi) (Chun et al., 2007). The sequences were also deposited in GenBank.

Yeast strain identification was performed using MALDI-TOF MS. Samples of cultures grown on Sabouraud agar plates (37°C, 72 h) were prepared using the ethanol/formic acid/acetonitrile extraction procedure (Bruker Daltonics, Bremen, Germany). Colony material was directly transferred and spotted onto an MBT Biotarget 96 target plate, air dried, and overlaid with 1 μL of a saturated α-cyano-4-hydroxycinnamic acid (HCCA) matrix solution in 50% of acetonitrile and 2.5% of trifluoroacetic acid. MALDI-TOF mass spectra for species identification and typing were generated on a MALDI Biotyper system (based on a Microflex LT/SH instrument; Bruker Daltonik) in a mass/charge (m/z) range of 2.000 to 20.000 with default settings for routine species identification. The system was calibrated daily using the IVD Bacterial Test Standard (Bruker Daltonik) as recommended by the manufacturer. Bruker Biotyper software, version 12.0, with the MBT IVD library containing 11.897 mass spectra was used.

### Quantitative assays for the characterization of plant growth−promoting traits

#### Characterization of nitrogen fixation potential: acetylene reduction test

The N_2_-fixing ability of all isolates was assessed *in vitro* using an indirect method that relies on the conversion of acetylene to ethylene (Stewart et al., 1967). This method, known as the acetylene reduction assay (ARA), involves incubating the strains in the presence of acetylene (C_2_H_2_), which is then reduced to ethylene (C_2_H_4_) by the bacterial nitrogenase enzyme. The rate of ethylene production in the ARA is indicative of the rate of nitrogen fixation by the bacterial strains. Thus, the ARA serves as a reliable proxy for assessing the nitrogen-fixing ability of the isolates.

In sterile 20 mL glass vials suitable for the Agilent 7679A Gas Chromatography Headspace Sampler, 100 µl of the bacterial isolates with a concentration of 3x10^8^ CFU.mL^-1^ was added to a volume 5 ml of Burks medium without N. The vials were then sealed with a nut clamp. A gas-tight syringe was used to remove 15 mL of air from the vials, which was replaced with an equivalent volume of acetylene. The vials were incubated at 30°C for 24 hours and analyzed for the presence of ethylene (C_2_H_4_) using Agilent 7890A gas chromatography with MS detection. The ethylene standard curve was determined by injecting known concentrations in order to correlate with the observed peaks. The results are expressed in mol C_2_H_4_.24h^-1^. Culture^-1^.

#### Quantitative determination of phosphate solubilization

The Phosphate solubilization ability of the isolates was assessed through both qualitative and quantitative tests, using two sources of inorganic phosphate: tri-calcium phosphate (Ca_3_ (PO_4_) ^2^) and naturel phosphate (NP). The quantitative analysis was carried out in Erlenmeyer flasks (250 mL) containing 50 mL of P (5 g.L^− 1^) mixed with PVK medium and inoculated with each of the isolate at approximately 3.10^8^ CFU.mL^− 1^. The flasks were then placed in a rotary shaker at 30°C and 150 rpm for a duration of 5 days. Control flasks with no inoculation were included as control for comparison. This experiment was performed in three repetitions. After 5 days of incubation, the pH of the medium was measured. The supernatant of each sample was obtained by centrifugation at 3800 rpm for 15 min, and the inorganic phosphate content was analyzed using an automated Continuous Flow Analyzer (CFA) (Skalar, Netherlands). This analysis was based on the molybdenum blue method, which involves a reaction between orthophosphate ions, ammonium heptamolybdate and potassium antimony (III) oxide tartrate to form the antimony-phosphomolybdate complex. This complex was then intensely reduced to a blue color by ascorbic acid. A standard curve was prepared using potassium dihydrogen phosphate (KH_2_PO_4_) in the range of 0.2; 0.4; 0.6; 0.8 and 1 mg P.L^− 1^.

#### Indole-3-acetic acid production assay

The production of IAA by nitrogen fixing strains was determined using a standard method (Loper and Schroth, 1986; Biswas et al., 2018). Each isolate was cultivated individually by adding 100 μL of overnight bacterial culture (10^7^ CFU.mL^−1^) to LB liquid medium containing 0.1 g.L^−1^ of l-Tryptophan. After incubation at 30°C in an orbital shaker (150 rpm for 3 days), each culture broth was centrifuged at 4000 rpm for 15 min, and the resulting supernatant was quantified spectrophotometrically at 535 nm using Salkowski reagent (comprising 0.5 M FeCl_3_:70% perchloric acid/water (2:49:49 ratio). The concentration of IAA (μg.mL^− 1^) in the cultures was estimated by comparing the readings to a standard curve using various concentrations of IAA (Fluka) (0; 5; 10; 20; 50 and 100 μg.mL^−1^).

### Plant growth and inoculation under gnotobiotic conditions

#### Wheat seed bacterization

The isolates were cultured overnight in 50 mL of Burk’s medium at 30°C with shaking in an orbital incubator shaker at 150 rpm. Bacterization of wheat seeds was prepared as follow: Wheat (*Triticum aestivum* L. var. KARIM) seeds were surface sterilized by chlorine gas, which consisted of a solution containing 3.5% sodium hypochlorite NaClO and 2.5% hydrochloric acid (HCl) for 15 min. After surface sterilization, the wheat seeds were rinsed with sterile distilled water and allowed to dry under sterile conditions. A solution of 0.5% (w/v) carboxymethyl cellulose (CMC) was prepared and mixed with bacterial cells. This mixture was applied to the wheat seeds under shaking until a fine coating was evenly distributed on seeds. The control consisted of cell-free seeds coated with CMC. The coated seeds were then examined to determine the number of bacterial cells per seed and were germinated on sterile paper at a temperature of 25°C for a duration of 2 to 3 days.

#### Wheat plant growth under gnotobiotic conditions

Both coated and uncoated grains were germinated for 2 to 3 days in the dark. Subsequently, uniform seedlings were placed in culture trays, with each tray containing 40 g of a sterile mixture of vermiculite and sand (in a 3:2 v/v ratio). Irrigation of the plants was carried out using a rotation of four applications with sterile distilled water followed by one application with Broughton nutrient solution (Broughton and Dilworth, 1970) based on the requirements of the specific treatment. The Broughton nutrient solution was N-free for the plants inoculated with bacteria and the negative control. While the positive control group received a nutrient solution containing N as an additional element. Each treatment was conducted in 6 replicates and placed in a phytotron under controlled conditions. These conditions included a temperature of 26°C, a photoperiod of 16 hours of light and 8 h of darkness, and a light intensity of 240 μmol photons m^-2^ s^-1^ for 20 days. At the end of the experiment, various growth parameters, including the height and weight of the plants, were determined for each treatment.

### Pot experiments

#### Nutrient levels

The experiments were conducted in pots measuring 12cm x 9.5cm, each containing 1 kg of a sand/vermiculite substrate mixture (3:2 v/v) sterilized in an autoclave. This substrate was intentionally made inert to control N fertilization levels for the wheat plants.

In this test, two sources of P were employed: Triple Super Phosphate (TSP) fertilizer and natural phosphate NP at a rate of 40 Kg/ha. These P sources were combined with three different levels of N in the form of Ammonium Nitrate: 0 kg/ha, 30 kg/ha and 60kg/ha.

#### Seed Inoculation

The strains selected from the previous assays were cultured in Burk’s medium at 30°C with shaking in an orbital incubator shaker at 150 rpm. The method of seed inoculation was carried out as previously described.

#### Plants harvest and growth study parameters assessment

The plants were harvested after 8 weeks of growth. The following parameters of the plants were evaluated: The dry weight and the height of both the aerial and root parts, the chlorophyll index, which was measured using the SPAD chlorophyll meter (SPAD-502 Plus; Konika Minolta) and finally the determination of the N and P content in the plant as follows: The entire plant was transferred to a digestion tube, and the digestion mixture (containing sulfuric acid and selenium) was added to the plant samples. The digestion tubes were then placed in a block digester preheated to 300°C. The P and N uptake by the plant in this assay were determined by analyzing the plant’s P and N content using the San+ Continuous Flow Analyzer (Skalar, The Netherlands).

### Statistical analysis

The statistical analysis and the determination of significant differences between the means of the various parameters studied were conducted using a two-factor ANOVA analysis with a significance level set at p<0.05. This analysis was performed using R software (version 4.1.2 - 2022-07-02). Each level of nitrogen was individually compared for the Pot experiments.

The principal component analysis (PCA) was carried out to explore the relationships and correlations between various variables. These variables included the rate of nitrogen fixation, phosphate solubilization, IAA production and plant growth indicators such as plant weight and height. The PCA was performed using R software.

## Results

### Isolation and molecular screening of nitrogen fixing strains

N_2_-fixing strains were isolated both from rhizospheric and non-rhizospheric soils using selective Burk’s media. In total, 472 isolates were obtained from the various soil samples. The results of the counts showed a variability in the number of N_2_-fixing bacteria in the collected soil samples. Some samples lacked populations of N_2_-fixing bacteria, while the highest recorded count was 8.5x10^6^ CFU.g^-1^ soil, as previously reported in Aasfar et al. (2021).

Following DNA extraction and the characterization of *nif-H* gene using the two pairs of primers, it was shown that the *nif-H* gene was present in 22 isolates among all the selected isolates. The presence of the *nif*-H gene in these isolates suggests their potential capability for nitrogen fixation.

### Confirmation of nitrogen fixation potential of *nif-H+* strains

Nitrogenase activities were assessed by measuring acetylene reduction activity for the screened strains. As presented in **Table 1**, the rates obtained from the strains fell within a range of 0.59 and 225.78 nmol C_2_H_4_.24h^-1^. culture^-1^ for NF 350 and NF 528 strains, respectively. Notably, strains NF 528, NF 524, NF 391, and NF 430 exhibited higher effectiveness in nitrogen fixation, with rates of 225.78; 138.88; 143.67; 183.88 nmol C_2_H_4_.24h-1. culture^-1^, respectively. These rates surpassed the reference strain *Azotobacter chroorcoccum* DSM2286, which had a rate of 114.21 nmol C_2_H_4_.24h^-1^. culture^-1^. On the other hand, some isolates showed lower levels of nitrogen fixation, including NF 350, NF 389, and NF 352, with rates of 0.59, 1.36, 2.36 nmol C_2_H_4_.24h^-1^. culture^-1^, respectively.

**Table 1:** *In vitro* plant growth promotion traits of the selected nitrogen fixing strains.

Strains	ARA (nmol C_2_H_4_.24h^-1^.culture^-1^)	Phosphate solubilization				IAA production (μg.mL^−1^)
		Natural phosphate (NP) (5 g.L^−1^)		Tri-calcium phosphate (TCP) (5 g.L^−1^)		
		(mg.L^−1^)	pH	(mg.L^−1^)	pH	
NF 63	15	200 ± 4.00^a^	3.56 ± 0.17	104 ± 0.00^cd^	5.47 ± 0.03	13.62 ± 0.02^lm^
NF 73	46.86	12 ± 1.00^l^	3.38 ± 0.05	81.5 ± 0.50^efg^	5.54 ± 0.06	22.82 ± 0.26^i^
NF 191	6.39	11.5 ± 1.50^i^	5.115 ± 0.12	91 ± 6.00^de^	5.52 ± 0.01	27.36 ± 0.10^h^
NF 197	7.3	109.5 ± 4.50^d^	4.92 ± 0.00	99 ± 6.00^cd^	4.67 ± 0.47	0.00 ± 0.00^p^
NF 229	4.3	30 ± 3.00^ij^	4.08 ± 0.02	103.5 ± 4.50^cd^	4.95 ± 0.09	23.06 ± 0.2^i^
NF 251	4.27	127 ± 1.00^c^	3.42 ± 0.14	53.5 ± 0.50^jk^	3.82 ± 0.01	8.53 ± 0.00^n^
NF 253	6.18	69.5 ± 4.50^fg^	3.72 ± 0.03	156 ± 8.00^a^	3.83 ± 0.02	13.96 ± 0.03^l^
NF 256	3.81	50.5 ± 0.50^h^	3.37 ± 0.04	77.5 ± 0.50^fg^	6.66 ± 0.61	36.83 ± 0.23^e^
NF 350	0.59	107.5 ± 2.50^d^	3.67 ± 0.07	133 ± 11.00^b^	4.08 ± 0.30	31.23 ± 0.3^g^
NF 352	2.36	10 ± 1.00^l^	4.76 ± 0.63	41 ± 2.00^kl^	6.02 ± 1.31	63 ± 1.53^a^
NF 389	1.36	23.5 ± 1.50^k^	3.86 ± 0.68	58 ± 7.00^ij^	4.21 ± 0.09	57.6 ± 0.20^b^
NF 391	143.66	27 ± 4.00^k^	4.68 ± 0.01	38 ± 0.00^l^	5.92 ± 0.14	36.46 ± 0.46^e^
NF 430	183.87	30.5 ± 1.50^ij^	5.01 ± 0.12	80 ± 4.00^efg^	5.66 ± 0.01	33.4 ± 0.53^f^
NF 468	85.99	28 ± 0.00^ij^	5.44 ± 0.37	76.5 ± 6.50^fg^	5.74 ± 0.10	2.83 ± 0.035°
NF 491	17.27	194 ± 0.00^a^	3.64 ± 0.40	107 ± 0.00^c^	5.27 ± 0.27	50.26 ± 2.40^c^
NF 511	14.28	169 ± 0.00^b^	3.60 ± 0.03	132 ± 0.00^b^	5.73 ± 0.00	1.36 ± 0.3^op^
NF 512	71.02	64.5 ± 5.50^g^	3.52 ± 0.01	81 ± 0.00^efg^	5.86 ± 0.02	19.7 ± 0.23^j^
NF 514	65.03	70 ± 0.00^fg^	4.13 ± 0.46	84 ± 0.00^ef^	5.88 ± 0.01	26.13 ± 0.26^h^
NF 515	88.63	36 ± 0.00^ij^	3.99 ± 0.48	63 ± 0.00^hij^	4.98 ± 0.62	46.83 ± 0.10^d^
NF 516	127.79	81.5 ± 6.50^e^	3.46 ± 0.01	76 ± 0.00^fgh^	5.75 ± 0.02	18.26 ± 0.86^jk^
NF 524	138.88	41 ± 0.00^i^	4.24 ± 0.85	72 ± 0.00^fgh^	5.04 ± 0.04	14.06 ± 0.26^l^
NF 528	225.77	73 ± 0.00^f^	3.35 ± 0.05	69.5 ± 7.50^ghi^	5.66 ± 0.05	11.73 ± 0.46^m^
*A. chroococcum DSM2286*	114.21	51 ± 4.00^h^	3.88 ± 0.30	139.5 ± 4.50^b^	5.53 ± 0.03	17.58 ± 0.01^k^

.*Evaluation of nitrogen fixation capabilities (using the acetylene to ethylene reduction test ARA), phosphorus solubilization (using two phosphorus sources: Moroccan Natural Phosphate NP, and Tricalcium Phosphate TCP), and indole-3-acetic acid (AIA) production in all 22 strains that tested positive for the nif-H gene.

.*Different letters represent significant statistical values at (p ≤ 0.05).

#### Quantitative determination of phosphate solubilization

The obtained *nif-H*+ strains were further screened to confirm their P solubilizing capacity. This was done through quantitative analysis in modified PVK liquid medium supplemented with different inorganic P sources. Almost all isolates demonstrated the ability to solubilize both commercial tricalcium phosphate (TCP) and Moroccan naturel rock phosphate to varying degrees.

**Table 1** presented the results, highlighting that strains NF 63, NF 491, NF 511 exhibited the highest level of Pi solubilization with values of 200 ± 4.00, 194 ± 0.00 and 169 ± 4.00 mg.L^−1^, respectively when using Moroccan NP. Conversely, the strain NF 352 achieved the lowest rate of Pi solubilization with Moroccan NP, at 10 ± 1.00 mg. L^−1^. In the case of TCP, the highest rate of Pi solubilization was achieved by strain NF 253, at 156 ± 8.00 mg. L^−1^, while strain NF 391 displayed the lowest rate, at 38 ± 0.00mg. L^−1^ (**Table 1**).

For some strains (NF 63, NF 197, NF 251, NF 491, NF 511, NF 516), solubilization of NP was more effective than TCP, which is a laboratory reagent. However, for the other strains, a lower solubilization rate was observed when they used NP as the source of inorganic phosphate in place of TCP. The process of P solubilization in liquid medium was accompanied by a decrease in pH.

#### Indole-3-acetic acid production assay

The quantification of indole-3-acetic acid production by the selected strains was conducted in the broth culture using Salkowski reagent supplemented with 0.1 g.L^−1^ of l-Tryptophan. The production of IAA was observed in almost all *nif-H*+ isolates, with values falling in the range of 1.36- 63.00 µg.mL^-1^. However, it’s worth noting that NF 197 did not produce IAA. Notably, strain NF 352 showed the highest rate of IAA production from l-Tryptophan among the screened strains with a production rate of 63.00 ± 1.53 µg.mL^-1^ (**Table 1**).

### Plant growth and inoculation under gnotobiotic conditions

The results obtained from the evaluation of the 22 *nif-H+* isolates showed that the removal of N from the nutrient irrigation solution had a significant negative impact on the growth parameters of the plants. This impact was most notable in the case of aerial biomass, which decreased by up to 50.58% (**Table 2**). However, the inoculation of wheat grains with the retained N_2_-fixing bacteria showed a substantial improvement in growth parameters (height and weight of the plants) compared to the control lacking N. The highest percentage increase in plant aerial biomass was 28.57% recorded with the strain NF 528, followed by the strain NF 516, which exhibited a 23.80% increase of plant growth parameters (**Table 2**).

**Table 2:** Effect of isolated nitrogen fixing isolates on growth parameters of wheat plants under gnotobiotic conditions.

Treatment	Plant Dw (mg)		Plant height (Cm)	
	Shoot	Root	Shoot	Root
Uninoculated control (-)^a^	42 ± 2.00 ^h^	22 ± 3.00 ^efg^	6.50 ± 0.36 ^hi^	15.93 ± 1.36 ^k^
Uninoculated control (+)^b^	85 ± 8.00 ^a^	26 ± 4.00 ^b-g^	10.13 ± 0.32 ^a^	24.70 ± 2.45 ^f-i^
NF 63	49 ± 4.00 ^b-g^	24 ± 5.00 ^c-g^	8.03 ± 0.15 ^efg^	30.33 ± 1.15 ^cd^
NF 73	51 ± 6.00 ^bc^	27 ± 7.00 ^b-g^	8.66 ± 0.35 ^de^	29.46 ± 1.76 ^cde^
NF 191	44 ± 2.00 ^e-h^	26 ± 2.00 ^c-g^	8.63 ± 0.11 ^de^	26.50 ± 0.50 ^d-h^
NF 197	43 ± 2.00 ^gh^	24 ± 10.00 ^d-g^	8.83 ± 0.28 ^cd^	37.80 ± 0.69 ^a^
NF 229	47 ± 3.00 ^b-h^	25 ± 3.00 ^c-g^	6.33 ± 0.20 ^hi^	23.40 ± 2.07 ^hi^
NF 251	45 ± 2.00 ^c-h^	26 ± 5.00 ^b-g^	6.66 ± 0.64 ^hi^	19.30 ± 3.31 ^jk^
NF 253	49 ± 3.00 ^b-g^	30 ± 6.00 ^a-d^	8.33 ± 0.32 ^def^	28.30 ± 0.30 ^c-f^
NF 256	50 ± 3.00 ^b-f^	24 ± 2.00 ^d-g^	7.83 ± 0.60 ^fg^	25.93 ± 2.45 ^e-h^
NF 350	50 ± 8.00 ^b-e^	28 ± 7.00 ^a-f^	8.23 ± 0.15 ^d-g^	21.16 ± 2.95 ^ij^
NF 352	47 ± 7.00 ^c-h^	31 ± 5.00 ^a-d^	6.10 ± 0.17 ^i^	24.36 ± 2.26 ^ghi^
NF 389	49 ± 3.00 ^b-f^	31 ± 3.00 ^abc^	9.33 ± 0.41 ^bc^	20.93 ± 0.40 ^ij^
NF 391	48 ± 3.00 ^b-h^	21 ± 2.00 ^fg^	9.53 ± 0.45 ^ab^	23.67 ± 2.89 ^hi^
NF 430	49 ± 3.00 ^b-f^	22 ± 2.00 ^efg^	9.76 ± 0.45 ^ab^	30.30 ± 0.80 ^cd^
NF 468	46 ± 8.00 ^c-h^	26 ± 5.00 ^c-g^	7.96 ± 0.41 ^fg^	31.90 ± 2.26 ^bc^
NF 491	50 ± 3.00 ^bcd^	20 ± 2.00 ^g^	8.30 ± 0.20 ^def^	29.86 ± 1.47 ^cd^
NF 511	44 ± 5.00 ^d-h^	29 ± 8.00 ^a-e^	8.26 ± 0.45 ^d-g^	35.06 ± 0.56 ^ab^
NF 512	43 ± 4.00 ^fgh^	20 ± 5.00 ^g^	6.96 ± 0.51 ^h^	25.50 ± 3.79 ^fgh^
NF 514	51± 7.00 ^bc^	28 ± 6.00 ^a-f^	8.13 ± 0.56 ^efg^	21.03 ± 1.32 ^ij^
NF 515	49 ± 5.00 ^b-f^	30 ± 2.00 ^a-d^	7.90 ± 0.40 ^fg^	28.13 ± 6.85 ^c-g^
NF 516	52 ± 5.00 ^bc^	35 ± 3.00 ^a^	8.26 ± 0.15 ^d-g^	27.06 ± 1.56 ^d-h^
NF 524	45 ± 3.00 ^c-h^	23 ± 4.00 ^efg^	9.60 ± 0.17 ^ab^	27.16 ± 2.11 ^d-h^
NF 528	54 ± 2.00 ^b^	33 ± 2.00 ^ab^	8.63 ± 0.80 ^de^	36.43 ± 0.87 ^a^
*A. chroococcum* DSM2286	48 ± 2.00 ^b-h^	30 ± 2.00 ^a-d^	7.63 ± 57.00 ^g^	25.25 ± 3.74 ^d-g^

*^a^Treatment without bacterial inoculation and devoid of nitrogen supply.

*^b^Treatment without bacterial inoculation and with normal nitrogen supply.

*Least significant difference (p≤ 5).

### Molecular identification of nitrogen fixing strains

The N_2_-fixing isolates were identified through the analysis of PCR-amplified 16S rDNA sequences (**Table 3**). The obtained sequences were deposited in GenBank and compared to sequences available in the NCBI database (**Table 3**).

**Table 3:** Identification of nitrogen fixing isolates using 16S rDNA sequencing.

Isolate ID	Genbank accession no	Related species	Accession no	Similarity %
NF 63	ON797666	*Pseudomonas brassicacearum CIP 109457*	NR_116299.1	99.47
NF 73	ON797675	*Bacillus pumilus CIP 52.67*	NR_115334.1	99.90
NF 191	ON797677	*Bacillus pumilus CIP 52.67*	NR_115334.1	98.29
NF 197	ON797678	*Pseudomonas chlororaphis DSM 6698*	NR_119340.1	98.61
NF 229	ON797681	*Bacillus pumilus CIP 52.67*	NR_115334.1	99.10
NF 251	ON797682	*Pseudomonas koreensis Ps 9-14*	NR_025228.1	98.01
NF 253	ON797683	*Pseudomonas koreensis Ps 9-14*	NR_025228.1	100.00
NF 256	ON814137	*Pseudomonas brenneri CFML 97-391*	NR_025103.1	99.39
NF 350	ON797690	*Pseudomonas koreensis Ps 9-14*	NR_025228.1	99.19
NF 352	ON860791	*Pseudomonas koreensis Ps 9-14*	NR_025228.1	99.19
NF 389	ON799424	*Burkholderia contaminans J2956*	NR_104978.1	99.90
NF 391	OR758906	*Pantoea brenneri* LMG 5343	NR_116748.1	97.76
NF 430	ON860821	*Pseudomonas lactis DSM 29167*	NR_156986.1	99.90
NF 468	ON804205	*Pseudomonas seleniipraecipitans CA5*	NR_116646.1	85.26
NF 491	ON804243	*Arthrobacter pascens DSM 20545*	NR_026191.1	96.43
NF 511	ON804343	*Pseudomonas frederiksbergensis JAJ28*	NR_028906.1	99.49
NF 512	ON804792	*Pseudomonas frederiksbergensis JAJ28*	NR_028906.1	99.59
NF 514	ON860823	*Bacillus wiedmannii FSL W8-0169*	NR_152692.1	99.70
NF 515	ON804886	*Bacillus subtilis IAM 12118*	NR_112116.2	99.80
NF 516	ON807311	*Rhodotorula mucilaginosa CBS*	NR_073296.1	99.81
NF 524	ON805794	*Pseudomonas frederiksbergensis JAJ28*	NR_028906.1	98.48
NF 528	ON862869	*Arthrobacter globiformis JCM 1332*	NR_112192.1	97.43

Among the screened strains, *Pseudomonas* emerged as the most frequently identified genus, with 12 strains falling into this genus, followed by the *Bacillus* genus which included 5 strains. Among the select isolates, NF 389 and NF 491 were affiliated with *Burkholderia* sp. and *Arthrobacter* sp, respectively. An interesting finding during this screening was the isolation of a yeast strain NF 516 which was subsequently identified using MALDITOF MS as *Rhodotorula mucilaginosa* with a score value of 2.09.

**Figure 1:**
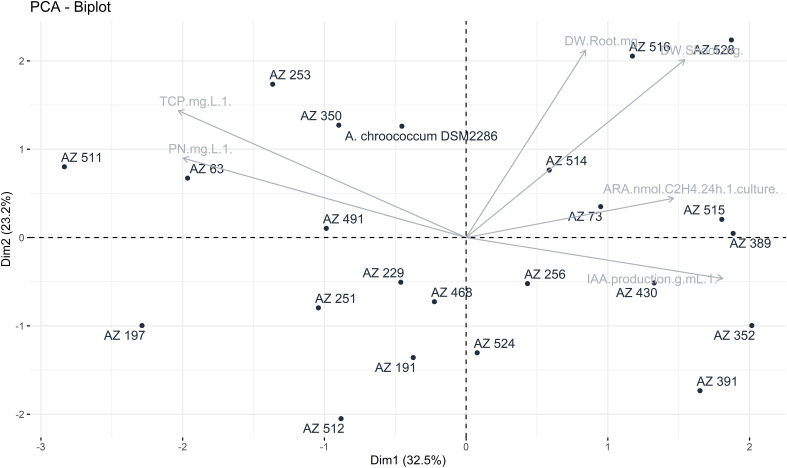
Principal component analysis between the following variables: nitrogen fixation rate, phosphate solubilization, IAA production and plant growth (weight and height).

To aid in the selection of promising isolates for pot assays, a two-dimensional graphical projection via Principal Component Analysis (PCA) was performed. All metabolic traits were correlated with each other using R software with the “FactorMiner” and “Corrplot” packages. The results, as shown in **Figure 1**, indicated that the plant and root weights were significantly correlated with variables related to the rate of acetylene reduction (ARA) and the production of IAA for the strains NF 528, NF 516 and NF 391. In light of these comprehensive findings, along with the data outlined in **Tables 1**, **2**, the strains R. *mucilaginosa* NF 516 and *Arthrobacter* sp. NF 528 emerge as promising candidates. Their demonstrated proficiency in nitrogen fixation and substantial impact on plant growth highlight their potential utility to reduce nitrogen fertilization rates. Therefore, these strains were retained for further investigation, particularly in conjunction with natural phosphate (NP) and triple superphosphate (TSP) as sources of phosphorus fertilization, in upcoming pot experiments.

### Pot experiment using promising nitrogen fixing microbial strains

In this section, the analysis of the interaction between bacterial strains, TSP (triple superphosphate), NP (natural phosphate), three levels of nitrogen fertilization, and the growth of wheat plants was performed in pots using inert substrate. The results presented in **Table 4** indicated a significant increase (p<0.05) in both root and shoot biomass for plants inoculated with N_2_-fixing bacteria (*R. mucilaginosa* NF 516 and *Arthrobacter* sp. NF 528) at nitrogen fertilization levels of 0%, 50% and 100%, in comparison to uninoculated plants.

**Table 4:** Effect of bacterization of wheat plants by nitrogen-fixing strains on shoot and root height (A). shoot and root dry weight (B) and chlorophyll content (C) under different nitrogen fertilization regimes using TSP and RP fertilizer as source of phosphate.

A												
Strains	TSP						RP					
	Shoot height (cm)			Root height (cm)			Shoot height (cm)			Root height (cm)		
	0%	50%	100%	0%	50%	100%	0%	50%	100%	0%	50%	100%
*R. mucilaginosa* NF 516	6.84 ± 0,99^a^	15.90 ± 1,54^a^	19.93 ± 0,86^b^	25.04 ± 2,46^a^	24.50 ± 3,35^a^	18.93± 1,63^b^	7.00 ± 1,09^bc^	13.86 ± 0,93^c^	16.80 ± 1,09^a^	28.86 ± 1,74^a^	24.39± 2,34^a^	25.13± 2,63^b^
*Arthrobacter* sp. NF 528	7.13 ± 0,99^a^	15.70 ± 0,99^a^	22.80 ± 1,38^a^	23.81± 2,21^a^	23.30 ± 3,55^a^	21.76± 1,41^ab^	7.46 ± 0,83^ab^	14.76 ± 0,79^b^	15.40 ± 1,61^b^	23.10 ± 2,64^b^	22.83± 1,20^ab^	24.80± 1,46^b^
NF 516-NF 528	3.36 ± 0,51^b^	14.43 ± 1,66^b^	19.60 ± 1,07^b^	8.43± 1,76^b^	24.93 ± 5,12^a^	23.40± 6,47^a^	7.86± 0,61^a^	15.93 ± 0,99^a^	13.93 ± 0,99^c^	22.83 ± 1,40^b^	22.20± 2,84^b^	27.03± 1,43^a^
Uninoculated control *	3.20 ± 0,33^b^	12.40 ± 1,08^c^	13.73 ± 1,43^c^	4.166 ± 1,266^c^	24.16 ± 6,15^a^	23.26± 4,29^a^	6.46± 0,66^c^	13.23 ± 0,88^c^	14.00 ± 1,66^c^	24.16 ± 3,23^b^	22.60± 3,37^ab^	22.40± 2,28^c^

B												
Strains	TSP						RP					
	Shoot Dw (g)			Root Dw (g)			Shoot Dw (g)			Root Dw (g)		
	0%	50%	100%	0%	50%	100%	0%	50%	100%	0%	50%	100%
*R. mucilaginosa* NF 516	0.120± 0,02^a^	0.973± 0,08^a^	1.779± 0,07^a^	0.074± 0,01^b^	0.880± 0,07^a^	1.675± 0,07^a^	0.097± 0,00^c^	0.671± 0,05^c^	1.189± 0,12^a^	0.068± 0,00^b^	0.860± 0,10^a^	0.909± 0,05^a^
*Arthrobacter* sp. NF 528	0.130± 0,01^a^	0.972± 0,10^a^	1.808± 0,35^a^	0.080± 0,01^a^	0.641± 0,07^b^	1.754± 0,09^a^	0.104± 0,00^b^	0.722± 0,05^b^	1.077± 0,12^b^	0.060± 0,00^c^	0.514± 0,05^c^	0.918± 0,10^a^
NF 516-NF 528	0.022± 0,00^b^	0.849± 0,07^b^	1.686± 0,25^a^	0.002± 0,00^c^	0.684± 0,05^b^	1.213± 0,21^b^	0.110± 0,00^a^	0.808± 0,05^a^	1.023± 0,11^b^	0.077± 0,01^a^	0.602± 0,02^b^	0.788± 0,09^b^
Uninoculated control *	0.012± 0,00^b^	0.606± 0,10^c^	1.046± 0,16^b^	0.001± 0,00^c^	0.506± 0,03^c^	0.867± 0,16^c^	0.078± 0,00^d^	0.571± 0,04^d^	0.898± 0,16^c^	0.072± 0,00^ab^	0.417± 0,01^d^	0.673± 0,10^c^

C												
Strains	TSP			RP								
	0%	50%	100%	0%	50%	100%						
*R. mucilaginosa* NF 516	21.35± 2,90^a^	45.79± 5,89^a^	45.00± 3,53^a^	21.82± 2,59^b^	42.58± 2,96^a^	38.57± 3,23^bc^						
*Arthrobacter* sp. NF 528	19.41± 3,21^a^	44.64± 3,68^a^	45.46± 4,42^a^	25.92± 2,84^a^	39.72± 4,43^b^	47.31± 3,37^a^						
NF 516-NF 528	5.03± 0,25^b^	45.84± 4,74^a^	46.26± 3,44^a^	26.28± 4,09^a^	37.90± 4,33^b^	40.34± 7,47^b^						
Uninoculated control *	6.30± 3,84^b^	32.74± 6,91^b^	36.70± 5,41^b^	12.38± 3,17^c^	34.08± 3,58^c^	35.15± 4,27^c^						

**Treatment without bacterial inoculation and with normal nitrogen supply.

.*Least significant difference (p ≤ 0.05).

Generally, the use of NP as a source of P fertilization resulted in a slight decrease in dry biomass produced by wheat plants compared to TSP. Specifically, when considering the highest value of aerial biomass for non-inoculated wheat plants, it was 1.046 ± 0.16 g under TSP fertilization, whereas it under NP fertilization, it was 0.898 ± 0.16 g (i.e. a decrease of 16.48%). This decrease could be explained by the soluble form of TSP unlike NP. On the other hand, no significant differences were found in plant height and chlorophyll content.

Under nitrogen-depleted conditions (0% N), individual bacterial strains demonstrated significant improvements in plant height, biomass, and chlorophyll content compared to the control. For plants fertilized with TSP, *R. mucilaginosa* NF 516 and *Arthrobacter* sp. NF 528 displayed interesting plant heights of 6.84 ± 0.99 cm and 7.13 ± 0.99 cm, respectively, in contrast to the 3.20 ± 0.33 cm observed in the control plants. Similarly, shoot biomass increased to 0.120 ± 0.02 g and 0.130 ± 0.01 g for NF 516 and NF 528, respectively, outperforming the control’s 0.012 ± 0.00 g. Under this nitrogen-free regime, the plants inoculated by the consortium (NF516 - NF 528) showed lower result compared to the use of the strains individually, with a height of 3.36 ± 0.51 cm and a shoot weight of 0.022 ± 0.00 g (**Table 4**).

In the presence of TSP and under a 50% nitrogen fertilization regime, the plants exhibited notable variations in height. Specifically, the inoculation with the strains *R. mucilaginosa* NF 516 and *Arthrobacter* sp. NF 528 displayed plant heights of 15.90 ± 1.54 cm and 15.70 ± 0.99 cm, respectively. These measurements significantly surpassed the control plant group with a mean height of 12.40 ± 1.08 cm. The inoculation with the consortium (NF 516 - NF 528) also contributed to this trend, with a plant height of 14.43 ± 1.66 cm, indicating the potential synergy between these bacterial strains in promoting plant growth under limited nitrogen conditions. Concerning the dry weight of the plant, inoculation with strains NF 516 and NF 528 reached an aboveground biomass of 0.973 ± 0.08 g and 0.972 ± 0.10 g, while the consortium (NF 516 - NF 528) recorded 0.849 ± 0.07 g, all surpassing the 0.606 ± 0.10 g of the control plants. Furthermore, under 100% nitrogen fertilization, the aerial biomass reached 1.779 ± 0.07 g with NF 516 inoculation, 1.808 ± 0.35 g with NF 528 and 1.686 ± 0.25 g with the consortium, compared to 1.046 ± 0.16 g for the control plants.

Under NP fertilization, even under 0% N conditions, wheat plant height surged 7.00 ± 1.09 cm, 7.46 ± 0.83 cm, and 7.86± 0.61 cm when the strains *R. mucilaginosa* NF 516, *Arthrobacter* sp. NF 528, and the consortium (NF 516 - NF 528) were applied, respectively, compared to the 6.46 ± 0.66 cm of the control. This trend also extended to shoot biomass, with NF 516, NF 528 and the consortium producing 0.097 ± 0.00 g, 0.104 ± 0.00 g and 0.110 ± 0.00 g, respectively, surpassing the 0.078 ± 0.00 g of the control plants. Under 50% nitrogen fertilization, aboveground biomass values of plants inoculated with strains NF 516, NF 528, and Consortium increased to 0.671 ± 0.05 g, 0.722 ± 0.05 g, and 0.808 ± 0.05 g, respectively. Under 100% nitrogen fertilization, values increased to 1.189 ± 0.12 g, 1.077 ± 0.12 g and 1.023 ± 0.11 g respectively, compared to 0.571± 0.04 g and 0.898 ± 0.16 g for the control plants.

**Figure 2:**
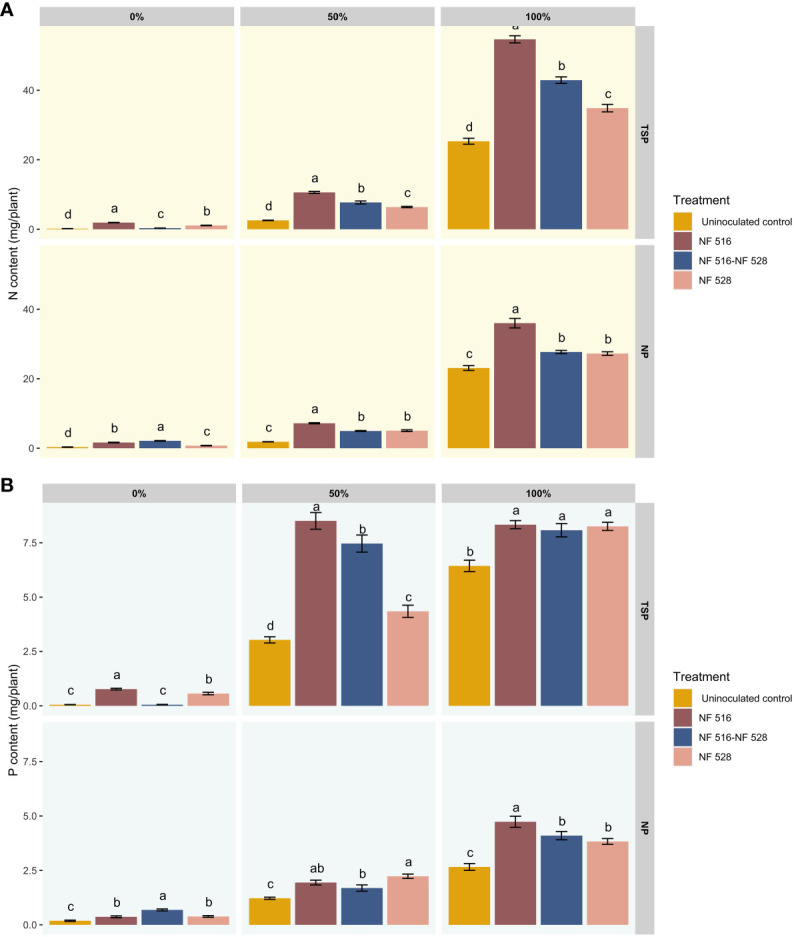
**(A)** Effect of bacterization of wheat plants by N2-fixing strains on the nitrogen contents of plants according to different nitrogen nutrition regimes and using TSP and NP as a source of phosphate fertilizer. **(B)** Effect of bacterization of wheat plants by N2-fixing strains on the phosphorus contents of plants according to different nitrogen nutrition regimes and using TSP and NP as a source of phosphate fertilizer.

**Figure 2** presents the results of N and P contents in inoculated and non-inoculated plants using an automated colorimetric method with a continuous flow analyzer (San++, Skalar). The findings demonstrated a highly significant effect of inoculation with *R. mucilaginosa* NF 516 and *Arthrobacter* sp. NF 528 on the N levels of plants across all the nitrogen levels used in this test, compared to control plants. At 0% N fertilization, the N contents were 1.90 ± 0.22 mg/plant, 1.09 ± 0.30 mg/plant and 0.31± 0.11 mg/plant for the plants inoculated with the strains *R. mucilaginosa* NF 516, *Arthrobacter* sp. NF 528 and the consortium (NF 516- NF 528), respectively. At 50% N fertilization, N contents were 10.6 ± 1.09 mg/plant, 6.38 ± 0.72 mg/plant and 7.70 ± 1.60 mg/plant. At 100% N fertilization, N contents were 54.65 ± 4.11 mg/plant, 34.84 ± 4.17 mg/plant and 42.90 ± 3.62 mg/plant compared to the uninoculated control plants with 0.16 ± 0.10 mg/plant, 2.54 ± 0.26 mg/plant and 25.31 ± 3.31 mg/plant (**Figure 2A**). The Results showed that inoculation induced an increase of more than 50% in N content in the plants at the vegetative stage after 8 weeks of cultivation, at all the N levels applied, compared to the control. The yeast strain *R. mucilaginosa* NF 516 exhibited a particularly impressive increase of 115.87% in N content on wheat plants.

Under NP fertilization and when compared to the effects of TSP, the stimulation observed in nitrogen uptake is relatively moderate. Nevertheless, the inoculation of specifically chosen N_2_-fixing bacteria, either individually or in consortium, significantly enhanced nitrogen content in wheat plants. This enhancement is particularly evident under the 0% nitrogen scenario, where only NP and N_2_ serve as the primary sources of these major elements for the plant, especially in an inert substrate such as vermiculite/sand. At 0% N fertilization, the N contents were 1.63 ± 0.32 mg/plant, 0.74 ± 0.28 mg/plant and 2.11 ± 0.35 mg/plant for the plants inoculated with the *R. mucilaginosa* NF 516, *Arthrobacter* sp. NF 528 and the consortium (NF 516- NF 528), respectively. At 50% N fertilization, N contents were 7.18 ± 0.61 mg/plant, 5.04 ± 0.97 mg/plant and 4.96 ± 0.48 mg/plant. At 100% N fertilization, N contents were 35.99 ± 5.29 mg/plant, 27.25 ± 1.97 mg/plant, and 27.69 ± 1.79 mg/plant, compared to the uninoculated control with 0.32 ± 0.26 mg/plant, 1.85 ± 0.15 mg/plant and 23.08 ± 2.67 mg/plant (**Figure 2A**).

The analysis of P contents in the plants after inoculation with the strains in combination with the TSP fertilizer showed a significant difference between the inoculated plants and the control plants, mainly under the 50% and 100% N fertilization regimes (**Figure 2B**). In this case the effect on P nutrition can be attributed to the impact of the inoculated strains on P assimilation, since the form contained in the TSP is readily available to the plants. The concentration of P increased further with the *R. mucilaginosa* NF 516 and Consortium treatments in the 50% and 100% nitrogen fertilization levels compared to the control, but no significant difference was found between these two levels within the same treatment. The results at the 0% N fertilization level confirmed the low contribution of the consortium to P assimilation, which was justified by the low biomass yield obtained by this treatment. The same observation holds for the use of NP, where stimulation of P uptake is relatively moderate due to the complexity of the substrate used. However, the assimilation of P increased in almost all the treatments at the three levels of N fertilization.

## Discussion

The challenge to identify diverse groups of diazotrophs with advantageous traits for enhancing plant growth offers the opportunity to reduce the use of chemical fertilizers and their associated expenses, while simultaneously improving soil health. Notably, integrating strains with a multitude of plant growth-promoting properties can contribute to increased crop productivity in an environmentally sustainable manner. N_2_-fixing bacteria belong to this group that play an important role in improving plant growth through nitrogen fixation, converting it into usable N compound, and employing various mechanisms such as P solubilization, phytohormone production and siderophore secretion (Bhattacharyya and Jha, 2012; Backer et al., 2018). In this work, we investigated the potential of microorganisms from Moroccan soils, collected from diverse regions, to fix N_2_ as a primary feature. Subsequently, their characteristics that could positively influence the growth of wheat plants were examined. A total of 472 strains were isolated on Burk’s selective medium, and all these isolates underwent DNA extraction to confirm the presence of the *nif-H* gene within their genome using specific molecular screening tool. Promising strains were then identified using 16S rRNA sequencing approach.

A total of 22 strains were selected based on the presence of the *nif-H* gene, and their identification through 16S rRNA sequencing revealed a remarkable diversity among the isolated N_2_-fixing species. The most abundant species identified were *Pseudomonas* sp. followed by *Bacillus* sp. In addition, the presence of other species such as *Arthrobacter* sp. and *Burkholderia* sp. was noted. These species are already known to carry the *nif-H* gene (Oliveira et al., 1993; Gillis et al., 1995; Yan et al., 2008; Upadhyay et al., 2009; Da Silva et al., 2012; Sharon and Daniel, 2013; Li et al., 2017; Estrada et al., 2018; Liu et al., 2019; Koirala and Brozel, 2021). However, among the screened isolates, the isolate NF 516 was identified as a yeast affiliated with *Rhodotorula mucilaginosa* species. This yeast yielded a positive result in *nif-H* gene amplification, indicating its potential for N_2_ fixation. To the best of our knowledge, this in only the second report of nitrogen fixation by members of *R. mucilaginosa*. In a recent study by Paul et al. (2020), it was elucidated that the *nif-H* gene detected in PCR amplification of the strain ﻿*Rhodotorula mucilaginosa* JGTA-S1 belongs in fact to *P. stutzeri*, which forms a symbiotic relationship within this yeast. This unique partnership equips *R. mucilaginosa* with the capability to thrive and flourish in nitrogen-deficient conditions.

In this study, we analyzed the nitrogenase activity of various isolated strains (**Table 1**). The ARA is a well-established technique used to quantitatively measure the rate of N_2_ fixation (Kifle and Laing, 2016). In the ARA assay, the N fixation potential of each strain was assessed under *in vitro* conditions. Diazotrophs can reduce acetylene to ethylene (C_2_H_4_), a process equivalent to the conversion of the natural substrate N_2_ into ammonia by the nitrogenase enzyme (Yousuf et al., 2017). Thus, the ARA assay has been widely employed for the evaluation of BNF. The results showed that all isolates reduced acetylene to ethylene, but their activities varied. The highest nitrogenase activity (225.78 nmol C_2_H_4_.24h^-1^ culture-^1^ nmol) was exhibited by *Arthrobacter* sp NF 528. These results align with several previous studies demonstrating that species like *Arthrobacter*, *Bacillus* and *Pseudomonas* strains were capable of effectively reducing acetylene using the ARA assay, indicating their N fixing abilities (Gtari et al., 2012; Ding et al., 2015; Kaushal and Kaushal, 2015; Li et al., 2017; Pham et al., 2017). To date, there have been no reports confirming the ability of *Rhodotorula* to fix N_2_ through the ARA assay. In our study, this strain displayed an interesting result in terms of acetylene reduction (127.79 nmol C2H4.24h-1. culture^-1^). This result supports the hypothesis put forth by Paul et al. (2020), explaining that *Rhodotorula mucilaginosa*’s ability to fix N_2_ stems from the symbiotic relationship with a bacterial strain capable of fixing N_2_ (Paul et al, 2020).

Phosphorus (P) is one of the most important nutrients for plants, and it has a significant impact on plant growth and development (Wang et al., 2009). In the soil, P often takes on a highly insoluble form, making it inaccessible for plant uptake. in this study, the selected strains were evaluated for their ability to solubilize P. For this purpose, two different sources of P, tricalcium phosphate (TCP) and natural phosphate (NP), were used. All the tested strains were able to solubilize P derived from TCP. The capacity to efficiently solubilize TCP in liquid medium has been reported by numerous studies (Swain et al., 2012; Islam et al., 2016; Li et al., 2017; Tang et al., 2020). However, when it came to NP, not all strains demonstrated the same efficiency in solubilizing NP as they did with TCP. This phenomenon is in line with previous research findings by Azaroual et al. (2020) and Kumari et al. (2008) and can be attributed to the intricate nature of natural rock phosphate, which contains a wide range of components (Rodríguez and Fraga, 1999). Phytohormones are considered fundamental compounds for plant growth and play a pivotal role in developmental processes such as cell division and root elongation. The most common phytohormone produced by PGPR is Indol-3-acetic acid (IAA), which can be produced *in vitro* by enriching the LB medium with its precursor, L-tryptophan (Duca et al., 2014). In our study, the quantitative assay of IAA production revealed that most isolates produced IAA, except for one rhizospheric soil isolate that was unable to produce this phytohormone. The production levels of IAA ranged from 1.36 to 63μg mL^−1^. These findings are consistent with existing literature that underscores the capacity of various PGPR strains, including *Bacillus* sp. and *Pseudomonas* sp, to produce IAA in the presence of l-Tryptophan (Torres-Rubio et al., 2000; Wahyudi et al., 2011; Kumar et al., 2014a; Moustaine et al., 2017).

Under *gnotobiotic* conditions, wheat plants were cultivated on an inert substrate, allowing the nutrients to be supplied directly from the provided nutrient solution or through the introduction of the selected *nifH*+ strains. In the present study, significant improvements were observed in various biometric parameters, including the height and weight of shoots and roots in wheat plants as a results of seed coating with selected isolates compared to uncoated control plants. the most substantial increase, amounting to 28.57% in aerial biomass, was recorded for strain *Arthrobacter* sp. NF 528 (**Table 2**). These results align with previous research that highlighted the positive impact of introducing effective bacterial strains on the promotion of wheat plant growth (Rana et al., 2011; Akhtar et al., 2013; Kumar et al., 2014b).

The yeast *Rhodotorula mucilaginosa* NF 516 demonstrated the ability to solubilize P, a trait that has been widely studied within this yeast. Previous studies by Mundra et al. (2011); Xiao et al. (2013) and Diya et al. (2019) have reported the P solubilization capabilities of *Rhodotorula mucilaginosa* members. Additionally, in our study, *Rhodotorula mucilaginosa* was able to solubilize NP in a manner comparable to TCP, which is not in line with the work of Narsian et al (2010), who reported that this yeast is more efficient at solubilizing TCP than NP. Furthermore, this yeast strain possesses the potential to produce phytohormones, including 3-indole acetic acid (Xin et al., 2009; Limtong et al., 2014). In this study, *Rhodotorula* was capable of producing IAA with levels reaching up to 18.26 μg mL^-1^. The combination of these attributes, including BNF, P solubilization and IAA production, allowed this yeast strain to have a positive effect on the growth of wheat plants under controlled conditions, resulting in a percentage increase of 23.81% in the dry weight of the plant. Encouragingly, analogous results of increased plant yield (rice) following inoculation with this yeast were also reported by Paul et al. (2020).

We examined the unique traits of N_2_-fixing bacteria and their effects on wheat plants growth using PCA. This method grouped three interconnected factors. N_2_-fixing capacity and IAA production were closely related to key growth parameters, including dry weight and plant height. However, the solubilization of P did not seem to impact plant growth at this growth stage. Based on these findings, two strains were selected to evaluate their potential in reducing the need for chemical N and how they behaved in combination with TSP fertilizer and NP as phosphate fertilization sources. Consequently, *Rhodotorula mucilaginosa* NF 516 and *Arthrobacter* sp. NF 528 were used as plant inoculants, both separately and in consortium.

The use of TSP and NP as a P source did not impact the ability of the isolates to promote plant growth. In fact, all treatments produced promising results in terms of growth parameters (dry weight, plant length and chlorophyll levels) when compared to the control plants under all N fertilization levels. However, when NP was used as the phosphate source, a slight decrease in wheat plant dry biomass was observed compared to those fertilized with TSP. This could be attributed to the characteristics of these phosphate sources, with NP being less soluble and nutrients becoming available more slowly for plant uptake (Biswas et al., 2022; Argotte-Ibarra et al., 2022). Despite this, the absence of significant differences in plant height and chlorophyll content suggested that plants may have adapted to these nutrient variations, optimizing their growth and physiological processes.

Apart from the limited growth observed in the combined use of *Rhodotorula mucilaginosa* NF 516 and *Arthrobacter* sp NF 528 under 0% N conditions with TSP fertilizer as the source of P, the presence of the selected N_2_-fixing species had a diverse impact on various growth parameters. Across different nitrogen fertilization levels and with two different P sources, these strains significantly enhanced plant biomass, increasing both root and shoot biomass under varying levels of nitrogen fertilization. Furthermore, their influence extended to critical growth parameters such as plant height, chlorophyll content and plant biomass, where the microbial strains have shown their ability to promote plant growth. In each treatment, we observed improvements in plant dry weight, height and chlorophyll content when compared to the non-inoculated control at the same N fertilization level. These results are consistent with the findings of Sandeep et al. (2009), who reported that *Pseudomonas aeruginosa* (PGPR) improved plant growth parameters under similar fertilization levels. Saber et al. (2012) and Manzoor et al. (2017) also documented increased wheat plant yields when N_2_-fixing or phosphate solubilizing bacteria were applied individually or in combination.

This study made an intriguing observation regarding significant plant growth responses, even when nitrogen levels were reduced to 50% of the plants need. When TSP fertilizer was used, wheat plants that were inoculated with R. *mucilaginosa* NF 516 and *Arthrobacteria* sp. AZ 528, exhibited remarkable increases in height, with a growth of 15.8% and 14.3%, respectively, compared to control plants receiving the standard 100% nitrogen supply. Importantly, these two strains produced biomass levels with 50% nitrogen fertilization that were statistically comparable to those of control plants receiving the full nitrogen supply (**Supplementary Figure S1A**). Furthermore, when using NP as the phosphorus source, these strains showed a significant increase in aboveground plant biomass. Notably the consortium of the two strains resulted in biomass levels that were similar to those of the regularly fertilized control, even with only 50% nitrogen fertilization (**Supplementary Figure S1B**). This emphasizes the potential of these strains to be used as bio-inoculants, reducing the nitrogen requirements for optimal wheat plant growth during this vegetative stage. Similar trends were observed by Adesemoye et al. (2009), where plant growth parameters with PGPR treatment combined with 80% or 70% fertilizer were statistically equivalent to those with 100% fertilizer without PGPR. Similarly, Tennakoon et al. (2019) reported a significant 33% reduction in nitrogen fertilizer use without any accompanying reduction in yield (Adesemoye et al., 2009; Tennakoon et al., 2019).

Several studies have reported that inoculation with N_2_-fixing bacteria increased nutrient uptake and enhanced wheat growth (Abbasdokht and Gholami, 2010; Baldani et al., 2014; Hussain et al., 2016). However, the potential of *R. mucilaginosa* as a bioinoculant to reduce chemical fertilizers inputs and promote plant growth has remained relatively unexplored (Oliveira et al., 2019; Paul et al., 2020). In our study, all treatments involving bacterial, or yeast inoculation led to a significant increase in both N and P content in plant biomass compared to the uninoculated control plants. Notably, *R. mucilaginosa* NF 516, both alone and in combination with *Arthrobacter* sp. NF 528, resulted in higher levels of N and P in the plants. This impact was more pronounced when using TSP as a source of P. Similar trends were observed in wheat when supplementing 75% of the recommended fertilizer rate with a mixture of PGPR and AMF (arbuscular mycrorrhizal fungi) inoculants, resulting in plant growth, yield, and nutrient (N and P) uptake that were statistically equivalent to the full fertilizer rate without inoculants (Adesemoye et al., 2009). A similar increase in N yield (144%) was also observed in banana (*Musa acuminata Colla*) plantlets inoculated with PGPR (El-Kholy et al., 2005; Baset et al., 2010). Under nitrogen-free conditions, plant nitrogen assimilation could primarily be attributed to BNF processes facilitated by the inoculated bacteria. Similarly, under conditions where only 50% of nitrogen fertilization was applied, our study observed that the remaining nitrogen needs of the wheat plants could be fulfilled through bacterial BNF processes. However, under normal N fertilization conditions, an additional mechanism could be at play, enhancing nutrient assimilation by the plants.

## Conclusions

In this study, we confirmed the potential of N_2_-fixing species to enhance plant growth and nutrient “N & P” uptake. This enhancement can be attributed to various factors, including nitrogen fixation, phosphorus solubilization, phytohormones production, or the combined effects of these traits. We examined 22 isolates for these characteristics and their effect on wheat plants growth under controlled conditions. Subsequently, a single bacterial strain and a yeast were meticulously selected to investigate their ability to reduce the need for nitrogen fertilizers, their compatibility with two different sources of phosphorus, and their capacity to coexist without antagonism. The results demonstrated that all the combinations tested improved plant growth and nutrient assimilation, particularly for N and P. Therefore, the use of plant growth-promoting species as an environmentally friendly solution to enhance plant growth is a promising solution, and the inoculation of such strains could serve as valuable biofertilizers in soils, reducing the need of chemical fertilizers. Surprisingly, the identification of the yeast strain *Rhodotorula mucilaginosa* NF 516 within the study was an unexpected discovery, particularly the present study succeeded to describe this species (as a N_2_-fixing) for the second time following up works by Paul et al. (2020). Our results not only validate its ability to fix N but also highlight its efficacy in promoting the growth of wheat plants, primarily through nitrogen fixation, which confirms the multifaceted plant growth-promoting potential of *Rhodotorula mucilaginosa* NF 516, making it a valuable candidate for further agricultural applications. The mechanisms responsible for this biological fixation by this yeast remain a subject of ongoing research, requiring further studies to fully understand its potential and its use as a novel type of biofertilizer.

## Data availability statement

The data presented in the study are deposited in the NCBI repository, accession numbers are included in **Table 3**.

## Author contributions

AA: Writing – review & editing, Writing – original draft, Visualization, Validation, Software, Resources, Methodology, Investigation, Formal analysis, Data curation, Conceptualization. IM: Writing – review & editing, Validation, Supervision, Project administration, Methodology, Investigation. SA: Writing – review & editing, Software, Resources, Methodology. SL: Writing – review & editing, Methodology, Investigation. NE: Writing – review & editing, Resources, Methodology. AB: Writing – review & editing, Supervision, Methodology, Investigation. YZ: Writing – review & editing, Funding acquisition. AH: Writing – review & editing, Supervision.
