# Supplementary material for: Agronomic advantage of bacterial biological nitrogen fixation on wheat plant growth under contrasting nitrogen and phosphorus regimes
Source: Front Plant Sci. 2024 May 8;15:1388775. doi: 10.3389/fpls.2024.1388775 (PMC11109382; doi:10.3389/fpls.2024.1388775)
Supplement: Supplementary file 1 [file Image_1.pdf]

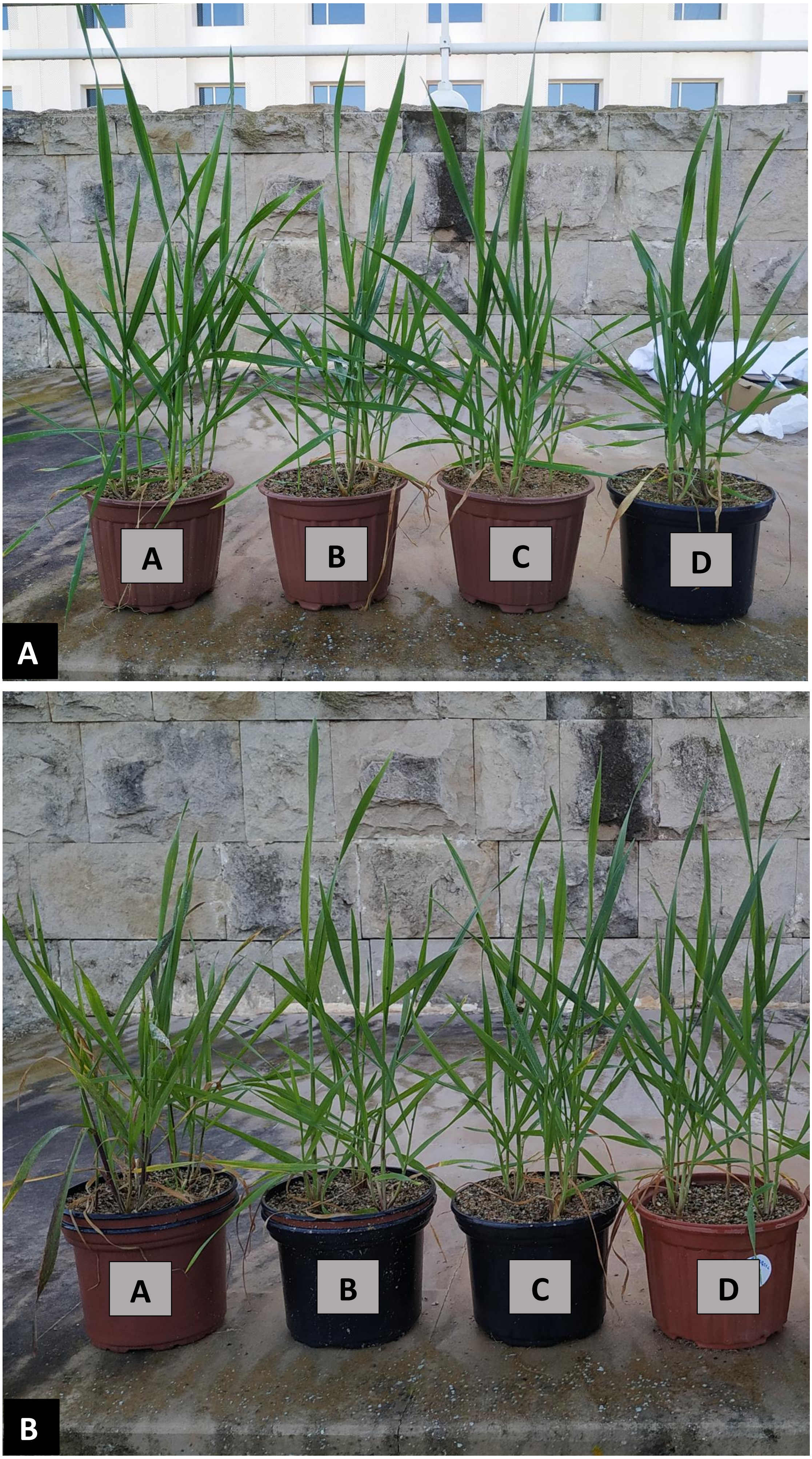
A
B
C
D
A
A
B
C
D
B

Fig. S1: Wheat Cultivation Experiment Comparing Different Bacterial Strains under Reduced Nitrogen Fertilization. In Fig S1.a, TSP was used as a phosphorus source, while in Fig S1.b, rock phosphate was used. The images illustrate the impact of utilizing various bacterial strains with 50% of nitrogen fertilization in comparison to the uninoculated control receiving 100% nitrogen fertilization.

(A) Uninoculated control at normal nitrogen fertilization (100%).
(B) Strain NF 516 at 50% of nitrogen fertilization.
(C) Consortium NF516-NF528 at 50% of nitrogen fertilization.
(D) Strain NF 528 at 50% of nitrogen fertilization.
