# Supplementary material for: Agronomic advantage of bacterial biological nitrogen fixation on wheat plant growth under contrasting nitrogen and phosphorus regimes
Source: Front Plant Sci. 2024 May 8;15:1388775. doi: 10.3389/fpls.2024.1388775 (PMC11109382; doi:10.3389/fpls.2024.1388775)
Supplement: Supplementary file 2 [file Table_1.docx]

| **Sample Code** | **Collection date** | **Location name** | **Latitude** | **Longitude** | **Area** | **Culture** |
| --- | --- | --- | --- | --- | --- | --- |
| **Z1RS1** | 18/10/2017 | Biougra | 30,1864 | -9,4044 | Sousse Massa | Tomato rhizospheric soil |
| **Z1RS2** | 18/10/2017 | Biougra | 30,1864 | -9,4044 | Sousse Massa | Tomato rhizospheric soil |
| **Z1RS2'** | 18/10/2017 | Biougra | 30,1864 | -9,4044 | Sousse Massa | Tomato rhizospheric soil |
| **Z2RS1** | 19/10/2017 | Ait Amira | 30,2492 | -9,4971 | Sousse Massa | Tomato rhizospheric soil |
| **Z2RS2** | 19/10/2017 | Ait Amira | 30,2492 | -9,497 | Sousse Massa | Cucumber rhizospheric soil |
| **Z2RS3** | 19/10/2017 | Ait Amira | 30,2492 | -9,4971 | Sousse Massa | pipper rhizospheric soil |
| **Z3BS1** | 19/10/2017 | Ait Amira | 30,2492 | -9,497 | Sousse Massa | Bulk soil after zucchini cultivation |
| **Z4RS1** | 19/10/2017 | Ait Amira | 30,2492 | -9,4971 | Sousse Massa | Green bean rhizospheric soil |
| **Z5BS1** | 28/10/2017 | Ouled Ghanem | 32,8684 | -8,8524 | El Jadida | Uncultivated and unfertilized soil |
| **Z5BS2** | 28/10/2017 | Ouled Ghanem | 32,8684 | -8,8524 | El Jadida | Uncultivated and unfertilized soil |
| **Z5BS3** | 28/10/2017 | Ouled Ghanem | 32,8684 | -8,8524 | El Jadida | Uncultivated and unfertilized soil |
| **Z6RS1** | 08/12/2017 | Boukallou | 33,0319 | -7,6242 | Settat | Barley rhizospheric soil |
| **Z6BS1** | 08/12/2017 | Boukallou | 33,0319 | -7,6242 | Settat | Uncultivated and unfertilized soil |
| **Z6BS2** | 08/12/2017 | Boukallou | 33,0319 | -7,6242 | Settat | Uncultivated and unfertilized soil |
| **Z6BS3** | 08/12/2017 | Boukallou | 33,0319 | -7,6242 | Settat | Uncultivated and unfertilized soil |
| **Z6RS2** | 08/12/2017 | Boukallou | 33,0319 | -7,6242 | Settat | Wheat rhizospheric soil |
| **Z7RS1** | 08/12/2017 | Route ouled Said | 33,0319 | -7,6241 | Settat | Mint rhizospheric soil |
| **Z7BS1** | 08/12/2017 | Route ouled Said | 33,0319 | -7,6241 | Settat | Wheat rhizospheric soil |
| **Z7BS2** | 08/12/2017 | Route ouled Said | 33,0319 | -7,6241 | Settat | Carrot rhizospheric soil |
| **Z7RS2** | 08/12/2017 | Route ouled Said | 33,0319 | -7,6241 | Settat | Carrot rhizospheric soil |
| **Z8BS1** | 08/12/2017 | Route de Marrakech / Khémiss Chaouia | 32,9185 | -7,6635 | Settat | Wheat rhizospheric soil |
| **Z8RS1** | 08/12/2017 | Route de Marrakech / Khémiss Chaouia | 32,9185 | -7,6635 | Settat | Alfalfa rhizospheric soil |
| **Z8RS2** | 08/12/2017 | Route de Marrakech / Khémiss Chaouia | 32,9185 | -7,6635 | Settat | Wheat rhizospheric soil |
| **Z8RS3** | 08/12/2017 | Route de Marrakech / Khémiss Chaouia | 32,9185 | -7,6635 | Settat | Olive tree rhizospheric soil |
| **Z9BS1** | 08/12/2017 | Route de marrakech / INRA Settat | 32,8505 | -7,749 | Settat | Uncultivated and unfertilized soil |
| **Z9BS2** | 08/12/2017 | Route de marrakech / INRA Settat | 32,8505 | -7,749 | Settat | Uncultivated and unfertilized soil |
| **Z9RS1** | 08/12/2017 | Route de marrakech / INRA Settat | 32,8505 | -7,749 | Settat | Carrot rhizospheric soil |
| **Z10RS1** | 28/12/2017 | Sidi Ayachi (El Gharb) | 34,3402 | -6,4437 | Kénitra | Sugar beet rhizospheric soil |
| **Z10RS2** | 28/12/2017 | Sidi Ayachi (El Gharb) | 34,3402 | -6,4437 | Kénitra | Alfalfa rhizospheric soil |
| **Z10RS3** | 28/12/2017 | Sidi Ayachi (El Gharb) | 34,3402 | -6,4437 | Kénitra | Wheat rhizospheric soil |
| **Z10RS4** | 28/12/2017 | Sidi Ayachi (El Gharb) | 34,3402 | -6,4437 | Kénitra | Barley rhizospheric soil |
| **Z10RS5** | 28/12/2017 | Sidi Ayachi (El Gharb) | 34,3402 | -6,4437 | Kénitra | Rhubarb rhizospheric soil |
| **Z11BS1** | 28/12/2017 | Douar El Khilate | 34,5268 | -6,3055 | Sidi Allal Tazi | Bulk soil of sugar cane cultivation |
| **Z11RS1** | 28/12/2017 | Douar El Khilate | 34,5268 | -6,3055 | Sidi Allal Tazi | Sugar cane rhizospheric soil |
| **Z11BS2** | 28/12/2017 | Douar El Khilate | 34,5268 | -6,3055 | Sidi Allal Tazi | Bulk soil of corn |
| **Z11RS2** | 28/12/2017 | Douar El Khilate | 34,5268 | -6,3055 | Sidi Allal Tazi | Rice rhizospheric soil |
| **Z12RS1** | 12/01/2018 | Commune Haouzia, conté chouiraf | 33,1504 | -8,478 | El Jadida - Sidi Bennour | Bean rhizospheric soil |
| **Z12RS2** | 12/01/2018 | Commune Haouzia, conté chouiraf | 33,1504 | -8,478 | El Jadida - Sidi Bennour | Wheat rhizospheric soil |
| **Z12RS3** | 12/01/2018 | Commune Haouzia, conté chouiraf | 33,1504 | -8,478 | El Jadida - Sidi Bennour | Potato rhizospheric soil |
| **Z12BS1** | 12/01/2018 | Commune Haouzia, conté chouiraf | 33,1504 | -8,478 | El Jadida - Sidi Bennour | Uncultivated and unfertilized soil |
| **Z12RS4** | 12/01/2018 | Commune Haouzia, conté chouiraf | 33,1504 | -8,478 | El Jadida - Sidi Bennour | Potato rhizospheric soil |
| **Z12BS2** | 12/01/2018 | Commune Haouzia, conté chouiraf | 33,1504 | -8,478 | El Jadida - Sidi Bennour | Bulk soil of corn cultivation |
| **Z13RS1** | 12/01/2018 | village de Sidi Ismail | 33,059 | -8,4349 | El Jadida - Sidi Bennour | Barley rhizospheric soil |
| **Z13RS2** | 12/01/2018 | village de Sidi Ismail | 33,059 | -8,4349 | El Jadida - Sidi Bennour | Cabbage crop rhizospheric soil |
| **Z13RS3** | 12/01/2018 | village de Sidi Ismail | 33,059 | -8,4349 | El Jadida - Sidi Bennour | Fennel rhizospheric soil |
| **Z13RS4** | 12/01/2018 | village de Sidi Ismail | 33,059 | -8,4349 | El Jadida - Sidi Bennour | Wheat rhizospheric soil |
| **Z13RS5** | 12/01/2018 | village de Sidi Ismail | 33,059 | -8,4349 | El Jadida - Sidi Bennour | Vineyard rhizospheric soil |
| **Z13RS6** | 12/01/2018 | village de Sidi Ismail | 33,059 | -8,4349 | El Jadida - Sidi Bennour | Sugar beet rhizospheric soil |
| **Z13RS7** | 12/01/2018 | village de Sidi Ismail | 33,059 | -8,4349 | El Jadida - Sidi Bennour | Bean rhizospheric soil |
| **Z14RS1** | 24/01/2018 | BABE MERZOUKA | 34,2138 | -4,1205 | TAZA | Peas rhizospheric soil |
| **Z14RS2** | 24/01/2018 | BABE MERZOUKA | 34,2138 | -4,1205 | TAZA | Barley rhizospheric soil |
| **Z14RS3** | 24/01/2018 | BABE MERZOUKA | 34,2138 | -4,1205 | TAZA | Faba bean rhizospheric soil |
| **Z15RS1** | 24/01/2018 | Oued Amlil | 34,1831 | -4,2998 | TAZA | Onions rhizospheric soil |
| **Z15RS2** | 24/01/2018 | Oued Amlil | 34,1831 | -4,2998 | TAZA | Chickpea rhizospheric soil |
| **Z15RS3** | 24/01/2018 | Oued Amlil | 34,1831 | -4,2998 | TAZA | Bean rhizospheric soil |
| **Z15RS4** | 24/01/2018 | Oued Amlil | 34,1831 | -4,2998 | TAZA | Wheat rhizospheric soil |
| **Z16BS1** | 24/01/2018 | Bouhlou | 34,1319 | -4,428 | TAZA | Fallow land bulk soil |
| **Z16RS1** | 24/01/2018 | Bouhlou | 34,1319 | -4,428 | TAZA | Faba bean rhizospheric soil |
| **Z16RS2** | 24/01/2018 | Bouhlou | 34,1319 | -4,428 | TAZA | Lentil rhizospheric soil |
| **Z17RS1** | 25/01/2018 | Oued Inanen | 34,2033 | -4,801 | TAOUNATE | Faba bean rhizospheric soil |
| **Z18RS1** | 25/01/2018 | Tounate | 34,5004 | -4,6592 | TAOUNATE | Calendula rhizospheric soil |
| **Z18RS2** | 25/01/2018 | Tounate | 34,5004 | -4,6592 | TAOUNATE | Peas rhizospheric soil |
| **Z18RS3** | 25/01/2018 | Tounate | 34,5004 | -4,6592 | TAOUNATE | Red bell pepper rhizospheric soil |
| **Z19RS1** | 16/02/2018 | Ait Ourir | 31,5801 | -7,655 | El Haouz | Faba bean rhizospheric soil |
| **Z19RS2** | 16/02/2018 | Ait Ourir | 31,5801 | -7,655 | El Haouz | Peas rhizospheric soil |
| **Z19RS3** | 16/02/2018 | Ait Ourir | 31,5801 | -7,655 | El Haouz | Peas rhizospheric soil |
| **Z19RS4** | 16/02/2018 | Ait Ourir | 31,5801 | -7,655 | El Haouz | Faba bean rhizospheric soil |
| **Z19RS5** | 16/02/2018 | Ait Ourir | 31,5801 | -7,655 | El Haouz | Faba bean rhizospheric soil |
| **Z19RS6** | 16/02/2018 | Ait Ourir | 31,5801 | -7,655 | El Haouz | Faba bean rhizospheric soil |
| **Z19RS7** | 16/02/2018 | Ait Ourir | 31,5801 | -7,655 | El Haouz | Faba bean rhizospheric soil |
| **Z19RS9** | 16/02/2018 | Ait Ourir | 31,5801 | -7,655 | El Haouz | Barley rhizospheric soil |
| **Z19RS10** | 16/02/2018 | Ait Ourir | 31,5801 | -7,655 | El Haouz | Wheat rhizospheric soil |
| **Z19RS11** | 16/02/2018 | Ait Ourir | 31,5801 | -7,655 | El Haouz | Faba bean rhizospheric soil |
| **Z20RS1** | 16/02/2018 | Tamazouzte | 31,5801 | -7,655 | El Haouz | Faba bean rhizospheric soil |
| **Z20RS1** | 16/02/2018 | Tamazouzte | 31,5801 | -7,655 | El Haouz | Faba bean rhizospheric soil |
| **Z20RS2** | 16/02/2018 | Tamazouzte | 31,5801 | -7,655 | El Haouz | Alfalfa rhizospheric soil |
| **Z20RS3** | 16/02/2018 | Tamazouzte | 31,5801 | -7,655 | El Haouz | Clover rhizospheric soil |
| **Z20RS4** | 16/02/2018 | Tamazouzte | 31,5801 | -7,655 | El Haouz | Turnip rhizospheric soil |
| **Z20RS5** | 16/02/2018 | Tamazouzte | 31,5801 | -7,655 | El Haouz | Barley rhizospheric soil |
| **Z20RS6** | 16/02/2018 | Tamazouzte | 31,5801 | -7,655 | El Haouz | Tomato rhizospheric soil |
| **Z20RS7** | 16/02/2018 | Tamazouzte | 31,5801 | -7,655 | El Haouz | Strawberry rhizospheric soil |
| **Z20RS8** | 16/02/2018 | Tamazouzte | 31,5801 | -7,655 | El Haouz | Faba bean rhizospheric soil |
