# Supplementary material for: Agronomic advantage of bacterial biological nitrogen fixation on wheat plant growth under contrasting nitrogen and phosphorus regimes
Source: Front Plant Sci. 2024 May 8;15:1388775. doi: 10.3389/fpls.2024.1388775 (PMC11109382; doi:10.3389/fpls.2024.1388775)
Supplement: Supplementary file 3 [file Table_2.docx]

**Table: Morphological characteristics of nitrogen-fixing nif-H+ screened strains.**

| **Strain code** | **Gram** | **Colony morphology** | **Cell morphology** |
| --- | --- | --- | --- |
| NF 63 | + | Round, regular, convex | Bacilli |
| NF 76 | - | Round, regular, umbonate | Cocco-bacilli |
| NF 191 | (-)+ | Round, Irregular, Flat | Bacilli |
| NF 197 | (-)+ | Round, regular, umbonate, rough | Bacilli |
| NF 229 | - | Round, regular, convex | Bacilli |
| NF 251 | - | Round, regular, raised | Bacilli |
| NF 253 | - | Round, regular, convex, smooth | Bacilli |
| NF 256 | - | Round, regular, convex, smooth | Bacilli |
| NF 350 | - | Round, regular, convex, smooth | Bacilli |
| NF 352 | - | Round, undulate, raised | Cocco-bacilli |
| NF 389 | - | Irregular, raised, rough | Bacilli |
| NF 391 | - | Round, regular, convex | Cocco-bacilli |
| NF 430 | - | Round, regular, Flat | Bacilli |
| NF 468 | - | Round, regular, umbonate | Bacilli |
| NF 491 | (-)+ | Round, regular, raised | Bacilli |
| NF 511 | - | Irregular, lobate, raised | Bacilli |
| NF 512 | - | Round, lobate, raised | Bacilli |
| NF 514 | - | Irregular, convex, rough | Bacilli |
| NF 515 | - | Round, regular, raised | Bacilli |
| NF 516 | - | Round, lobate, raised | Bacilli |
| NF 524 | + | Round, regular, convex | Cocco-bacilli |
| NF 528 | (-)+ | Round, regular, convex | Bacilli |
